# Histone Deacetylase Inhibitor Treatment Increases the Expression of the Plasma Membrane Ca^2+^ Pump PMCA4b and Inhibits the Migration of Melanoma Cells Independent of ERK

**DOI:** 10.3389/fonc.2017.00095

**Published:** 2017-05-24

**Authors:** Luca Hegedüs, Rita Padányi, Judit Molnár, Katalin Pászty, Karolina Varga, István Kenessey, Eszter Sárközy, Matthias Wolf, Michael Grusch, Zoltán Hegyi, László Homolya, Clemens Aigner, Tamás Garay, Balázs Hegedüs, József Tímár, Enikö Kállay, Ágnes Enyedi

**Affiliations:** ^1^Department of Thoracic Surgery, Ruhrlandklinik, University Clinic Essen, Essen, Germany; ^2^Department of Pathophysiology and Allergy Research, Comprehensive Cancer Center Vienna, Medical University of Vienna, Vienna, Austria; ^3^2nd Institute of Pathology, Semmelweis University, Budapest, Hungary; ^4^Molecular Biophysics Research Group of the Hungarian Academy of Sciences, Department of Biophysics, Semmelweis University, Budapest, Hungary; ^5^MTA-SE-NAP Brain Metastasis Research Group of the Hungarian Academy of Sciences, Semmelweis University, Budapest, Hungary; ^6^Department of Medicine I, Institute of Cancer Research, Comprehensive Cancer Center Vienna, Medical University of Vienna, Vienna, Austria; ^7^Institute of Enzymology, Research Centre for Natural Sciences, Hungarian Academy of Sciences, Budapest, Hungary; ^8^Molecular Oncology Research Group of the Hungarian Academy of Sciences, Semmelweis University, Budapest, Hungary

**Keywords:** PMCA4, PMCA1, HDAC inhibitors, cell motility, BRAF-mutant melanoma

## Abstract

Several new therapeutic options emerged recently to treat metastatic melanoma; however, the high frequency of intrinsic and acquired resistance among patients shows a need for new therapeutic options. Previously, we identified the plasma membrane Ca^2+^ ATPase 4b (PMCA4b) as a metastasis suppressor in BRAF-mutant melanomas and found that mutant BRAF inhibition increased the expression of the pump, which then inhibited the migratory and metastatic capability of the cells. Earlier it was also demonstrated that histone deacetylase inhibitors (HDACis) upregulated PMCA4b expression in gastric, colon, and breast cancer cells. In this study, we treated one BRAF wild-type and two BRAF-mutant melanoma cell lines with the HDACis, SAHA and valproic acid, either alone, or in combination with the BRAF inhibitor, vemurafenib. We found that HDACi treatment strongly increased the expression of PMCA4b in all cell lines irrespective of their BRAF mutational status, and this effect was independent of ERK activity. Furthermore, HDAC inhibition also enhanced the abundance of the housekeeping isoform PMCA1. Combination of HDACis with vemurafenib, however, did not have any additive effects on either PMCA isoform. We demonstrated that the HDACi-induced increase in PMCA abundance was coupled to an enhanced [Ca^2+^]_i_ clearance rate and also strongly inhibited both the random and directional movements of A375 cells. The primary role of PMCA4b in these characteristic changes was demonstrated by treatment with the PMCA4-specific inhibitor, caloxin 1c2, which was able to restore the slower Ca^2+^ clearance rate and higher motility of the cells. While HDAC treatment inhibited cell motility, it decreased only modestly the ratio of proliferative cells and cell viability. Our results show that in melanoma cells the expression of both PMCA4b and PMCA1 is under epigenetic control and the elevation of PMCA4b expression either by HDACi treatment or by the decreased activation of the BRAF-MEK-ERK pathway can inhibit the migratory capacity of the highly motile A375 cells.

## Introduction

Metastatic melanoma is a highly invasive tumor type of the skin with poor prognosis. Although several new therapeutic options have emerged, the five-year survival of melanoma patients is only around 15% and the number of cases steadily increases in Western countries (1). Approximately 50% of melanomas carry a BRAF and 15% an NRAS mutation causing the activation of the mitogen-activated protein kinase (MAPK) pathway (2). In more than 90% of the BRAF-mutant melanoma cases, the activating mutation is V600E in the BRAF kinase. Specific inhibitors against V600E-mutant BRAF with low cross-reactivity toward wild-type BRAF and CRAF (3), vemurafenib and dabrafeniba initiated quick responses in a high percentage of patients; however, resistance emerged within 6–8 months in most of the cases (4). Inhibitors against MEK kinases were also developed, and the combination treatment of dabrafenib and the MEK inhibitor trametinib has been approved as a first-line therapy for patients with metastatic melanoma bearing BRAF V600E/K mutations (1). More recently, very promising results have been achieved with immune checkpoint inhibitors (5). In monotherapy, both CTLA-4 (ipilimumab) and PD-1 (nivolumab and pembrolizumab) inhibitors generated around 30–40% response rate, while in combination this was further increased to 60% (6). These response rates were independent of the BRAF mutational status of the tumor. Despite all of these advances, there remains a large group of patients who do not respond to either of these therapies or develop resistance quickly, requiring an ongoing search for new treatment options.

It has been known for a long time that histone acetylation is modified in cancer cells (7). Histone acetylation is controlled by the concerted action of histone acetyl transferases and histone deacetylases (HDACs) ultimately regulating the level of gene expression. HDAC inhibitors (HDACis) increase histone acetylation, leading to the activation of genes previously repressed or silenced in the tumor. They also have non-histone target proteins through which they influence other processes, such as apoptosis or angiogenesis (8). Three HDACis are already approved drugs in cancer therapy, suberoylanilide hydroxamic acid (SAHA, vorinostat) for cutaneous T-cell lymphoma and romidepsin and belinostat for peripheral T-cell lymphomas. Other inhibitors are tested in different tumor types such as breast (9), colon (10), pancreatic (11), and lung cancer (12). In melanomas, HDACis were mostly used in combination treatments in both *in vitro* experiments and clinical studies. In one study, high expression of HDAC 1, 2, and 3 was identified in all examined melanoma cell lines, while the expression of HDAC 4, 8, and 9 varied. HDAC expression seemed to be independent of the BRAF mutational status.

Upon combined treatment with the HDACi, SAHA, and the BRAF inhibitor, PLX4720, cell death was strongly induced in BRAF-mutant melanoma cells, while in melanocytes the treatment caused very low toxicity (13). Furthermore, in PLX4720-resistant cells, combination treatment with SAHA was able to overcome resistance and initiate cell death (13). It was also shown in melanomas that HDACis can decrease the expression of several anti-apoptotic proteins such as Bcl-XL or Bcl2, while they increase the expression of certain pro-apoptotic ones like BIM or BAX (14). However, there are substantial differences in the target spectrum of HDACis with distinct chemical structures. While SAHA belongs to the group of hydroxamic acids, another frequently used inhibitor, valproic acid, is a short-chain fatty acid. It was found that both SAHA and valproic acid were able to induce apoptosis in different melanoma cell lines, but they modulated apoptotic regulators differently (15).

Besides apoptosis induction, HDACis also increased immunogenicity of melanoma cells. Class I HDACi treatment increased PD-L1 and PD-L2 expression of melanoma cells (16), while pan-HDACis induced upregulation of antigen-presenting MHC molecules (17). Several phase I and II trials have been conducted with HDACis in combination with other agents; however, serious side effects and low response rates led to the discontinuation of most of these attempts (18). These uncertainties about HDACi action further increase the need to understand the molecular mechanisms behind HDACi treatments.

Plasma membrane Ca^2+^ ATPases (ATP2B; PMCA) are Ca^2+^ pumps, which are responsible for maintaining the low cytosolic Ca^2+^ concentration. Four genes (*ATP2B1-4*) encode for the PMCAs and alternative splicing of the primary transcript generates more than 20 isoforms (19). PMCA1 has a housekeeping role and it is found in every cell type. PMCA2 and 3 isoforms are found mostly in excitable cells, and a particular PMCA2 isoform (PMCA2wb) is found in the lactating mammary gland and at distinct regions of the brain. PMCA4b is also ubiquitous, and its expression was shown to be downregulated in colon and breast cancer cells (20). It was also demonstrated that after treatment with short-chain fatty acids or trichostatin A in colon cells, and SAHA and valproate in breast cancer cells, its expression was strongly upregulated (9, 10).

Recently, our group identified PMCA4b as a putative metastasis suppressor in melanoma (21). We reported that in BRAF-mutant melanoma cells, PMCA4b expression is specifically upregulated by the inhibition of the Ras–Raf–ERK pathway with BRAF or MEK inhibitors. Increased abundance of this pump resulted in enhanced Ca^2+^ clearance, and its overexpression reduced migration and metastatic activity of these cells *in vivo*.

It has been shown in several other publications that Ca^2+^ played an important role in the regulation of migration and metastasis of melanoma cells. The transient receptor potential cation channel subfamily M member 1 (TRPM1) Ca^2+^ channel was identified as a putative tumor suppressor in melanomas, and its expression negatively correlated with tumor aggressiveness (22). High levels of store-operated Ca^2+^ channel (ORAI calcium release-activated calcium modulator 1; ORAI1) and stromal interacting molecules 1 and 2 (STIM1 and 2) were described in melanoma cell lines and melanoma tissues. Inhibition of store-operated Ca^2+^ entry resulted in decreased migratory and metastatic activity of the cells (23, 24). It was also demonstrated that Ca^2+^ oscillations initiated by STIM1 and ORAI1 regulated invadopodium formation and proteolysis in melanoma cells (25).

In this study, we investigated the effect of HDACi treatment alone and in combination with the BRAF inhibitor, vemurafenib, on PMCA expression and on Ca^2+^ clearance in both BRAF-mutant and BRAF wild-type melanoma cell lines. While BRAF inhibition affected only the expression of the PMCA4b isoform, we found that SAHA and valproic acid treatment increased the abundance of both PMCA1 and PMCA4b. Combination of the HDACis with the BRAF-specific inhibitor had similar effect as either treatment alone. We also found that valproic acid treatment inhibited the migration of the highly motile A375 cells, and this effect could be partially reversed by the PMCA4b-specific inhibitor caloxin 1c2. Our work further supports the idea that PMCA4b can inhibit the migration and metastasis of melanoma cells when it is upregulated, and this effect is independent of the activation of the MAPK pathway.

## Materials and Methods

### Cell Culture

We used one BRAF/NRAS wild-type (MEWO) and two BRAF (V600E)-mutant (A375, A2058) melanoma cell lines. All the cell lines were purchased from ATCC and subjected to STR analysis at the Medical University of Vienna. We cultured the cells in DMEM supplemented with 10% FBS, 100 mg/ml streptomycin, 100 U/ml penicillin at 37°C, and 5% CO_2_ in a humidified atmosphere.

### Treatments of Cell Lines

Valproic acid (sodium salt; Sigma-Aldrich) was dissolved in distilled water at the concentration of 200 mM and stored at −20°C. From SAHA (Sigma-Aldrich), a stock solution was prepared at the concentration of 100 mM in DMSO and stored at −20°C. The mutant BRAF (V600E)-specific inhibitor, vemurafenib (PLX4032, Selleck Chemicals, Munich, Germany) was dissolved in DMSO (100 mM) and kept at −80°C. For Western blots, 1–2 × 10^5^ cells/well were seeded in 6-well plates. For Ca^2+^ signal measurements and immunofluorescence staining, 1–2 × 10^4^ cells/well were placed in an Imaging Chamber 8 Well (Ibidi). Twenty-four hours after seeding, medium was changed to fresh medium with the appropriate drug. For SAHA treatment, medium was changed daily. The final DMSO concentration did not exceed 0.01% in any of the experiments.

### Quantitative Real-time Reverse Transcription PCR

mRNA of several Ca^2+^ channels was isolated with TRIzol reagent (Life Technologies) from vemurafenib- and valproic acid-treated and control cells. For reverse transcription, RevertAid Reverse Transcriptase (Thermo Scientific) was used and amplification was performed with the Maxima SYBR Green master mix (Thermo Scientific) on an Applied Biosystems^®^ 7500 Real-Time PCR System. Primers used are described in Ref. (21).

### Western Blot Analysis

Total protein from the cells was precipitated by the addition of 6% TCA, and equal amounts of protein were loaded on 10% acrylamide gels. Samples were analyzed by Western blot as described previously (10). The following primary antibodies were applied: mouse monoclonal anti-PMCA4b (JA3, recognizing the region between residues 1,156–1,180, which is specific to hPMCA4b (26), dilution 1:1,000), anti-pan PMCA (5F10, dilution 1:5,000) (27), rabbit polyclonal anti-PMCA1 (Affinity BioReagents, PA1-914, dilution 1:1,000), mouse monoclonal anti-SERCA2 (IID8, dilution 1:2,500, Sigma-Aldrich, S1439), mouse monoclonal anti-SERCA3 [PL/IM430, dilution 1:200 (10)], rabbit monoclonal anti-phospho-p44/42MAPK (ERK1/2) (cell Signaling, CST4370S, dilution 1:1,000), mouse monoclonal anti-ERK1/2 (MK1) (Santa Cruz, sc135900, dilution 1:500), rabbit polyclonal anti-beta-tubulin (Abcam, ab6046), rabbit polyclonal acetyl-Histone H3 (Lys 9/Lys 14) (cell Signaling, 9677). Subsequently HRP-conjugated anti-rabbit and anti-mouse secondary antibodies (Jackson ImmunoResearch, dilution 1:10,000) were used, and detection was performed with Pierce ECL Western Blotting Substrate (Thermo Scientific) and luminography. Densitometric analysis was performed by ImageJ software v1.42q.

### Immunofluorescence

Cells were treated with 1.0 µM SAHA or 2 mM valproic acid for 48 h. First, the cells were washed twice with PBS at 37°C; then, they were fixed with 4% paraformaldehyde for 15 min at room temperature. Immunostaining experiments were performed as described previously (9). As primary antibody, mouse monoclonal anti-PMCA4b (JA3, dilution: 1:200) antibody was used. As secondary antibody, Alexa Flour 488-conjugated anti-mouse IgG (Invitrogen) was applied. Images were taken by a Zeiss LSM500 confocal laser scanning microscope.

### Ca^2+^ Signal Measurements

Cells were treated with 2.0 mM valproic acid for 48 h. In order to detect the intracellular Ca^2+^ level changes, the Fluo-4 green fluorescent Ca^2+^ indicator was used. Cells were kept in HBSS supplemented with 2 mM CaCl_2_, 0.9 mM MgCl_2_, and 20 mM HEPES pH7.4 during the entire experiment. First, cells were washed twice; then, they were incubated with 0.5 µM Fluo-4 AM (Molecular Probes, F14201) for 30 min at RT. After loading, cells were washed twice again. Ca^2+^ signals were induced by A23187 ionophore (2 µM). In order to specifically inhibit PMCA4 activity, cells were incubated with caloxin 1c2 (20 µM) for 10 min followed by the initiation of Ca^2+^ signaling with the addition of A23187. Time-lapse images were taken by Zeiss LSM500 and Olympus IX81 laser scanning confocal microscopes with a 60× (1.4) oil immersion objective. Z-resolution was set to 1 µm, and the images were taken every 1 s. The relative fluorescence intensities were calculated as F/F_o_ (where F_o_ was the average initial fluorescence), and the data were analyzed using the Prism4 software v4.01 (GraphPad Software).

### Non-Directional Cell Motility Assay

A375 cells were seeded at 1,000 cells per well in 96-well plates (Greiner). The next day, the cell nuclei were stained with 0.1 µM Hoechst 33342 for 1 h. The cells were imaged automatically with ImageXpress Micro XL (Molecular Devices, Sunnyvale, CA, USA) high content screening system using a Nikon CFI Super Plan Fluor ELWD ADM 10× objective. The cells were kept at 37°C in a humidified atmosphere containing 5% CO_2_ throughout the measurements. The fluorescence signals for Hoechst (447/60 nm) were acquired at 377/50 nm excitations. Four images per field with 2 × 2 binning were taken every 30 min for 24 h. Motility analysis was performed using the Multidimensional Motion Analysis module of MetaXpress High Content Image Acquisition and Analysis Software Version 5.3. The cell nuclei were analyzed in a size range of 9–15 µm. The maximum distance between two points on the trajectory of individual cells was set to 100 µm.

### Directional Cell Migration Assay

A modified Boyden chamber assay was previously described by Albini et al. (28). The assay was performed in a 48-well Boyden chamber (Neuro probe, Gaithersburg, MD, USA) on 10 μm-thick uncoated Nucleopore membranes (Whatman) with pore diameters of 8 µm. Fibronectin (100 µg/ml Millipore) was used as chemoattractant in the bottom chamber. Valproate pretreated (4 mM, 48 h) and untreated A375 melanoma cells were placed into the upper chamber at 2 × 10^4^ cells/well in DMEM containing 10% FCS. The 20 µM caloxin 1c2 was added as appropriate, and cells were allowed to migrate 6 h at 37°C. The filter was removed from the chamber, and the cells on the upper side of the filter were scraped off. The migrated cells on the lower side of the filter were fixed with methanol and stained with toluidine blue. Cell migration activity was quantified as the number of migrated cells on the lower side of the filter using a light microscope at ×200 magnification.

### Viability Assay

Viability was measured by the NucleoCounter NC-3000™ system (Chemometec). Cells were treated with 0.5 µM vemurafenib, 2 or 4 mM valproate, 1 µM SAHA alone, or in combination for 48 h in 6-well plates. After trypsinization, two dyes were added to the cells: acridine orange which stained all the cells in the solution and DAPI which was able to stain only the non-viable cells (Solution 13, Chemometec, 910-3013). 10 µl of each sample was loaded in an 8-well NC slide, and the counting of cells for the two cell populations was performed. Viability was calculated from the cell counts; viability = total cells − non-viable cells/total cells.

### Cell Cycle Analysis

The number of cells in each cell cycle phase was determined based on their DNA content. Cells were treated with 0.5 µM vemurafenib, 2 or 4 mM valproate, 1 µM SAHA alone, or in combination for 48 h in 6-well plates. After trypsinization, cells were treated with lysis buffer and stained with DAPI for 5 min at 37°C. Then, the stabilization buffer was added and 10 µl of each sample was loaded onto an 8-well NC slide. Cellular fluorescence was quantified by the NucleoCounter NC-3000™ system (Chemometec).

## Results

### HDACi Treatment Upregulates the Expression of PMCA1 and PMCA4b in Human Melanoma Cells Independent of the BRAF Mutational Status

**Figure 1:**
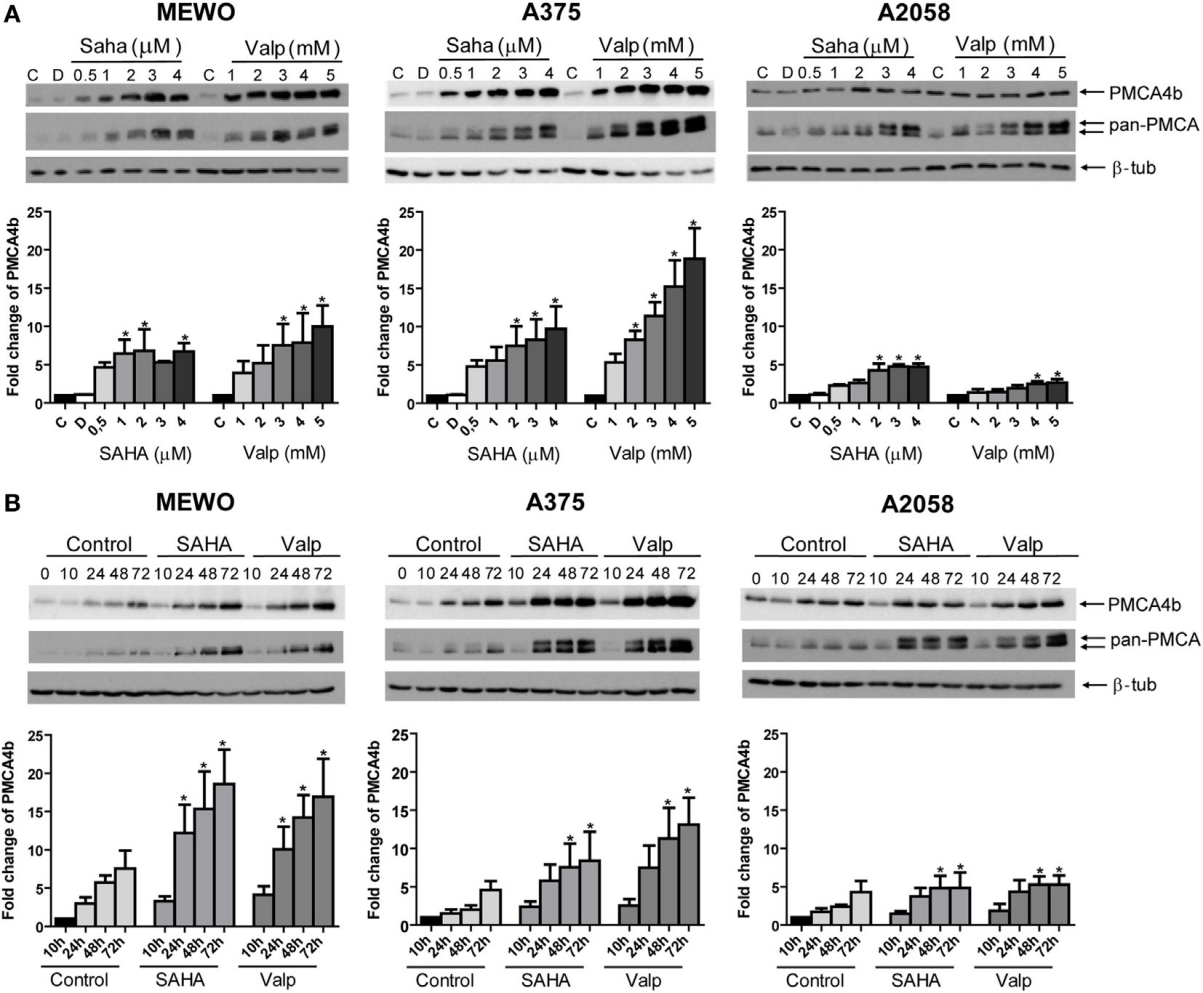
**PMCA4b is upregulated in both BRAF wild-type and BRAF-mutant melanoma cells after histone deacetylase inhibitor (HDACi) treatment**. **(A)** One BRAF wild-type (MEWO) and two BRAF-mutant (A375, A2058) cell lines were treated with HDACi SAHA or valproate in increasing concentrations for 48 h. DMSO (D) treatment was included as vehicle control. The protein level of PMCA4b and PMCA1 proteins were analyzed by Western blotting of total cell lysates (30 µg per sample) with PMCA4b-specific and pan-PMCA antibodies. Western blots were analyzed by densitometry. **(B)** Change in PMCA protein expression level was investigated after treatment with SAHA (2 µM) or valproate (2 mM) for 10, 24, 48, and 72 h with PMCA4b-specific and pan-PMCA antibodies. Western blots were analyzed by densitometry. Data were normalized to the expression levels of β-tubulin, and changes in PMCA4b protein level were expressed as fold increase over the untreated controls. Bars represent mean ± SE from three independent experiments. Asterisks indicate significant differences (*p < 0.05) from control, as calculated by ANOVA and Dunnett’s *post hoc* tests.

**Figure 2:**
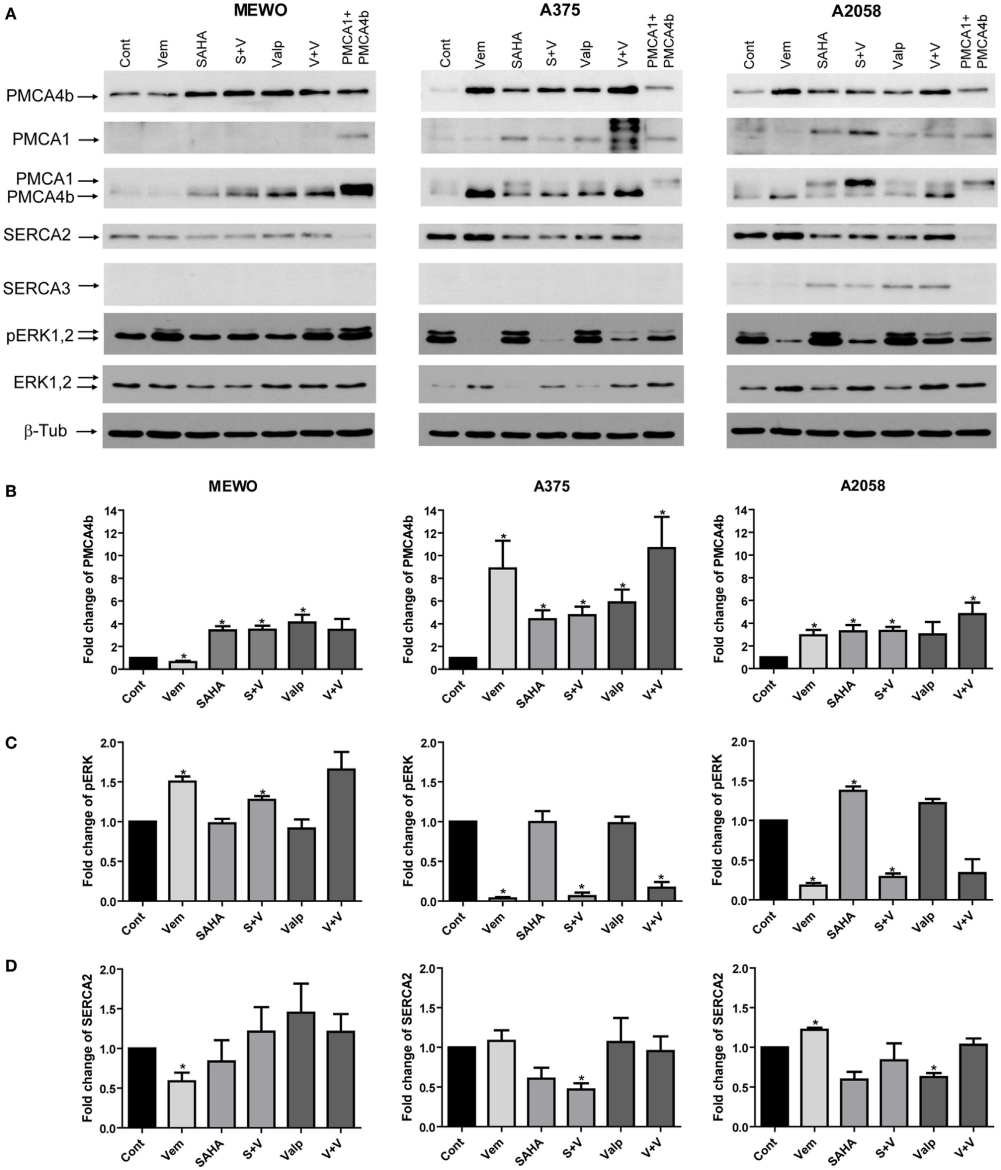
**Combination treatment with the mutant BRAF inhibitor vemurafenib and histone deacetylase inhibitors (HDACi)**. BRAF wild-type MEWO and BRAF-mutant A375 and A2058 cells were treated with the mutant BRAF inhibitor vemurafenib (0.5 µM), HDACi SAHA (1 µM), and valproate (2 mM) alone or in combination for 48 h. After Western blotting expression of PMCA4b, PMCA1, SERCA2, SERCA3, pERK, and ERK proteins were analyzed. As a positive control of PMCA1 and PMCA4b, total cell lysate of COS-7 cells was used, which endogenously express both proteins (9). **(A)** The expression of PMCA proteins was detected with isoform-specific and pan-PMCA antibodies. **(B)** After densitometric analysis, changes in PMCA4b **(B)**, ERK **(C)** and SERCA2 **(D)** protein levels were normalized to the expression levels of β-tubulin and expressed as fold increase over the untreated controls. Bars represent mean ± SE from three independent experiments. Significance compared to control is denoted by asterisks (*p < 0.05), using two-tailed paired *t*-test.

It was previously found that inhibition of HDAC activity increased PMCA4b expression in breast and colon cancer cell lines (9, 10). More recently, we demonstrated that in BRAF-mutant melanoma cells BRAF inhibitor treatment had a similar effect (21). To investigate how HDACis affected PMCA expression in melanoma cells, we treated one BRAF wild-type (MEWO) and two BRAF-mutant (A375, A2058) cell lines either with increasing concentrations of SAHA or valproate (Figure 1A) or for different time intervals (Figure 1B). We found that both HDACis strongly increased the expression of PMCA4b in MEWO and A375 cells and to a lesser extent in A2058 cells. Interestingly, in response to HDACi treatment, the pan-PMCA antibody, 5F10, also recognized a band just above the one corresponding to PMCA4b. The upper band could be identified as PMCA1 by using the PMCA1 isoform-specific antibody NR1 (Figure 2A). Both SAHA and valproate upregulated PMCA expression already at the lowest doses tested, 0.5 µM and 1 mM, respectively; half-maximal response was reached at about 1 µM concentration for SAHA and 2 mM for valproate. According to the time curve in Figure 1B, PMCA4b reached maximum abundance after 48 h. At the longer time points, a moderate increase in PMCA4b expression was seen also in the control samples, in good accordance with previous findings, indicating that PMCA4b expression increases with cell confluency (29).

### Combination Treatment of HDACis with Mutant BRAF Inhibitor Vemurafenib Is Not Additive

Combination of HDACis with targeted therapies for the treatment of melanoma is a possibility to overcome resistance (13). Since both HDAC and BRAF inhibition were able to increase PMCA4b expression in BRAF-mutant melanoma cells, we tested their combined effects. We treated two BRAF-mutant and one BRAF wild-type melanoma cell lines with the mutant BRAF inhibitor vemurafenib (0.5 µM) and with the HDACis SAHA (1 µM) and valproate (2 mM) alone, or in combination for 48 h (Figure 2). We found that in the BRAF-mutant A375 cell line vemurafenib had a stronger effect on PMCA4b abundance than the HDACis, and this was not further increased by combination with valproate or SAHA, while in A2058 cells HDACi treatment and BRAF inhibition had a similar effect and combination treatment with valproate had a slightly stronger effect than either drug alone (Figure 2B). In the BRAF wild-type MEWO cells, vemurafenib had no effect and combination treatments were similar to that of the HDACis alone, as expected. In good accordance with our previous results, PMCA1 abundance was not influenced by the BRAF inhibitor, vemurafenib, treatment; however, it was increased substantially by HDAC inhibition. Importantly, the staining with the antibody pair pERK/ERK clearly illustrates that HDACi treatment did not reduce ERK activation in any of the cell lines, while vemurafenib strongly decreased ERK phosphorylation in the BRAF-mutant cells (Figure 2C). In the BRAF wild-type MEWO cells, pERK level was slightly increased by the BRAF inhibitor treatment, which is in accordance with previous findings (30). This suggests that HDACi treatment increases PMCA abundance in an ERK-independent manner. To verify that HDACi treatment increased histone protein acetylation, we compared the acetylation level of histone 3 protein among control and HDACi-treated cells (Figure S3 in Supplementary Material). We found that both SAHA and valproate treatment increased H3 acetylation levels in all the cell lines in line with previous findings (31). In contrast, vemurafenib treatment did not enhance H3 acetylation in these cells.

We also tested the effect of HDAC inhibition alone or in combination with vemurafenib on PMCA4b expression at the mRNA level (Figure S1 in Supplementary Material). Cells were treated with valproate (2 mM) alone or in combination with vemurafenib (0.5 µM) for 48 h. In MEWO cells, PMCA4b expression was enhanced already after 8 h and further increased up to 48 h but then started to decline. In the A375 cells, the mRNA level of PMCA4b strongly increased after 24 h treatment and also started to decline after 48 h. In A2058 cells also PMCA4b mRNA level increased after 24 h but this increase was less substantial, except in the combination treatment, which induced strong increase.

### HDACis Alone or in Combination with the BRAF Inhibitor Vemurafenib Only Moderately Affected the Expression of Other Components of the Ca^2+^ Signaling Toolkit

The sarco/endoplasmic reticulum Ca^2+^ ATPases (SERCAs) are also important regulators of intracellular Ca^2+^ homeostasis, and the SERCA3 isoform was shown to be modified in cancer. Therefore, we analyzed the protein levels of SERCA2 and SERCA3, as well (Figure 2). We found that before treatment only SERCA2 was present in the melanoma cell lines and its protein level moderately decreased in response to HDACis in the BRAF-mutant cells (Figure 2D). SERCA3 expression, which is a known differentiation marker (32, 33), was induced by HDACi treatment in A2058 cells but was not detected in the other two cell lines.

To assess the effect of HDAC inhibition on Ca^2+^ channels—the “on” mechanisms of the Ca^2+^ signaling toolkit—we analyzed their mRNA expression before and after valproate treatment alone, or in combination with vemurafenib (Figure S2 in Supplementary Material). We found that in MEWO and A375 cells none of the treatments affected the expression of the inositol 1,4,5-trisphosphate receptor type 1–3 (IP3R1, IP3R2, IP3R3), the ORAI1, the stromal interaction molecule 1 and 2 (STIM1, STIM2), and the TRPM1. In A2058 cells, the mRNA level of IP3R3 was substantially increased by valproate, whereas TRPM1 expression was induced only by the combination treatment. Previously, we found that vemurafenib induced TRPM1 expression in this cell line suggesting that the increase was caused by the BRAF inhibitor in the combination treatment.

### HDACi Treatment Increased Plasma Membrane Abundance of PMCA4b in both Wild-Type and BRAF-Mutant Melanoma Cells

**Figure 3:**
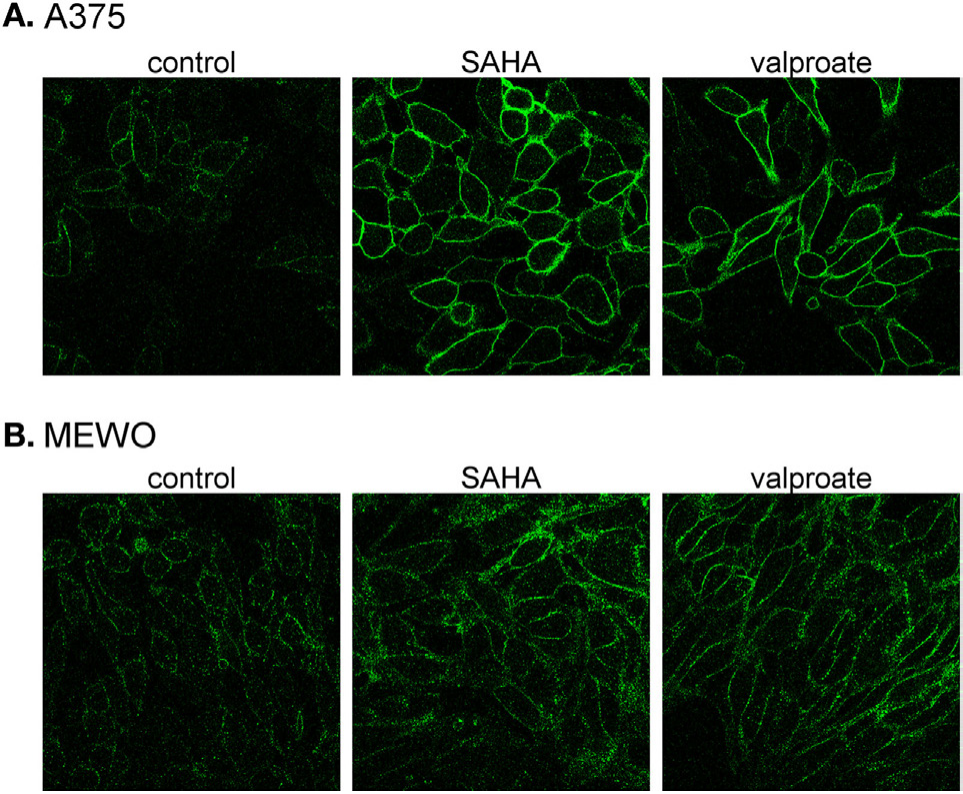
**Histone deacetylase inhibitor treatment increases PMCA4b abundance in the plasma membrane**. **(A)** BRAF-mutant A375 and **(B)** BRAF wild-type MEWO cells were treated with 1 µM SAHA, or 2 mM valproate for 48 h; then, the intracellular localization of PMCA4b was analyzed by immunofluorescence staining with anti-PMCA4b antibody (JA3). Images were taken by confocal microscope with a 60× objective.

We treated one BRAF wild-type (MEWO) and one BRAF-mutant (A375) cell line with the HDACis, SAHA (1 µM), or valproate (2 mM), for 48 h and then used confocal microscopy to analyze the intracellular localization of PMCA4b (Figure 3). We found that HDAC inhibition strongly increased the abundance of PMCA4b in the plasma membrane of both cell lines.

### Enhanced Ca^2+^ Clearance Initiated by both BRAF and HDACi Treatment Can Be Partially Reverted by Cotreatment with PMCA4b-Specific Inhibitor, Caloxin 1c2, in A375 Cells

**Figure 4:**
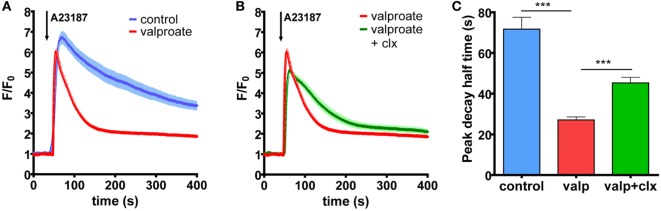
**Histone deacetylase inhibitor treatment increases Ca^2+^ clearance in BRAF-mutant A375 cells through PMCA4b activity**. **(A)** Ca^2+^ signaling measurement was performed after 48 h of treatment with 2 mM valproate. External media were changed to HBSS supplemented with 2 mM Ca^2+^, and cells were filled with intracellular Ca^2+^ indicator, Fluo-4. The Ca^2+^ signal was initiated by 2 µM A23187. **(B)** The PMCA4-specific inhibitor, caloxin 1c2 (20 mM) was added 10 min before stimulation with A23187 to the valproate-treated cells. Data represent fluorescent intensity values of 10–15 cells. **(C)** Half-peak decay time of the A23187-induced Ca^2+^ signal was determined after treatment with valproate alone or in combination with the PMCA4 inhibitor, caloxin 1c2, compared to control. Bar graphs are mean ± SD of individual cells taken from two to three independent experiments. Significance between control and valproate- or vemurafenib-treated cells is denoted by ***p < 0.001; two-tailed unpaired *t*-test.

We found previously that the vemurafenib-induced increase in PMCA4b abundance resulted in a faster Ca^2+^ clearance from the cells after stimulation. In order to assess whether HDACis had a similar effect, we measured intracellular Ca^2+^ signaling in A375 cells after valproate treatment with confocal imaging (Figure 4A). The Ca^2+^ signal was evoked by the Ca^2+^ ionophore A23187 that initiates Ca^2+^ uptake independently of the Ca^2+^ entry machinery. The Ca^2+^-sensitive fluorescent Fluo-4 indicator was used to monitor the changes in intracellular Ca^2+^ concentrations. For each measurement, we acquired the data with identical acquisition parameters and settings. We found that the Fluo-4 fluorescence signal declined to the basal level much faster after the peek in the valproate treated cells than in the control cells. Furthermore, this effect could be reverted to a significant extent by the addition of the PMCA4-specific inhibitor, caloxin 1c2, which inhibited the decay phase of the Ca^2+^ signal (Figures 4B,C). These results show that PMCA4b is primarily responsible for the increased Ca^2+^ clearance after HDACi treatment in these cells.

### The Effect of HDACi Treatment Alone and in Combination with BRAF Inhibition on Cell Viability and Cell Cycle Progression

**Figure 5:**
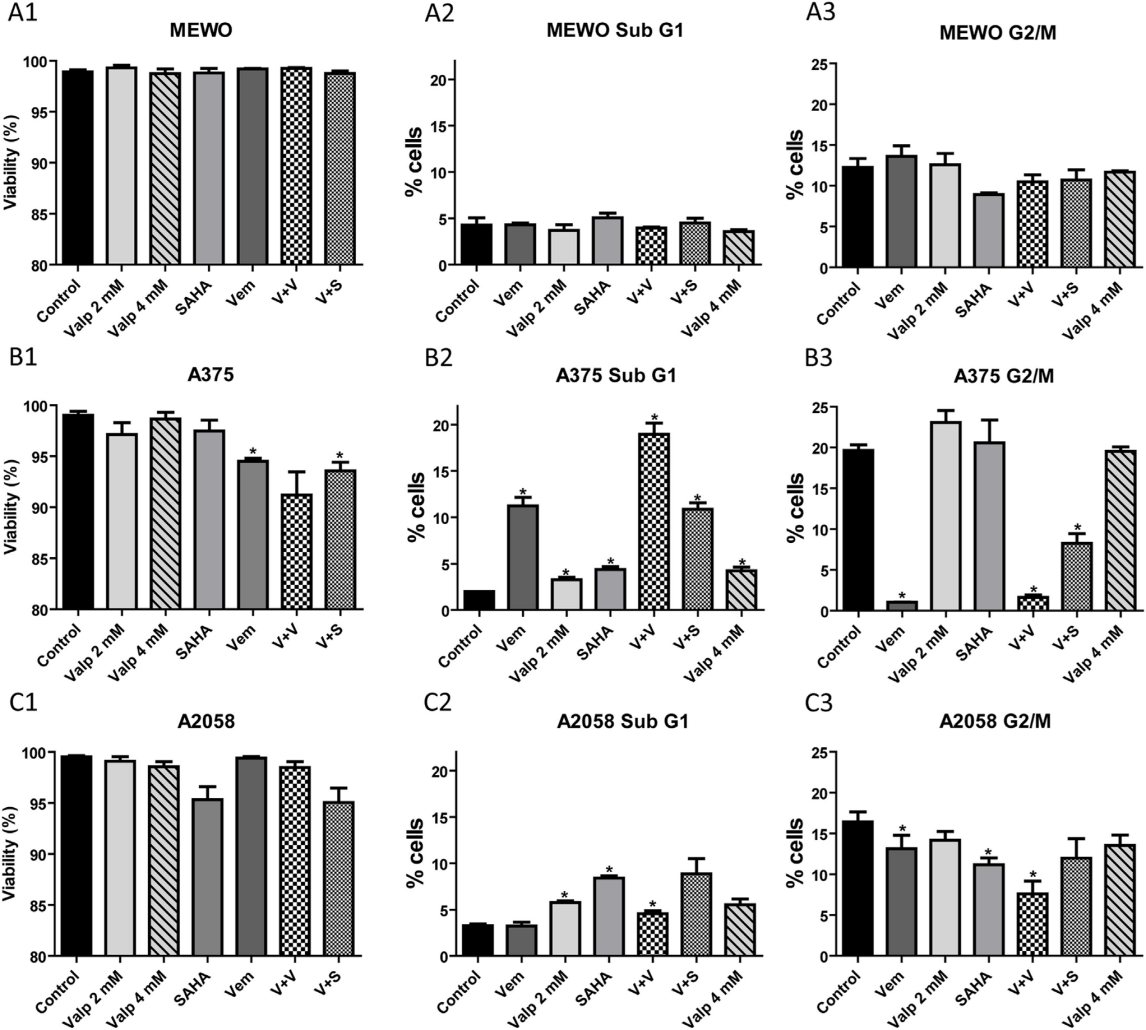
**Analysis of viability and cell cycle after treatment with histone deacetylase inhibitors alone and in combination with the BRAF inhibitor, vemurafenib**. Cells were treated with SAHA (1 µM), valproate (2 or 4 mM), and vemurafenib (0.5 µM) alone or in combination for 48 h. **(A)** The percentage of viable cells was determined by counting the number of total and non-viable cells. **(B)** The ratio of cells in sub-G1 and G2/M cell cycle phases was determined based on the cell’s DNA content. Data are means of three independent determinations. Significance compared to control is denoted by asterisks (*p < 0.05); two-tailed paired *t*-test.

Histone deacetylase inhibition was previously shown to induce apoptosis and to decrease proliferation in a variety of cancer cells. Furthermore, the combination of BRAF and HDACis increased cell death and even overcame resistance in BRAF-mutant melanoma cells (13). We treated the three melanoma cell lines (MEWO, A2058, and A375) with HDAC and BRAF inhibitors alone and in combination for 48 h and tested their viability (Figure 5). We found that none of the treatments affected significantly the viability of the BRAF wild-type MEWO cells, and BRAF-mutant A2058 cells (Figures 5A1,C1). In the A375 cells, vemurafenib alone or in combination with SAHA decreased the cell viability slightly, but significantly (Figure 5B1).

We also analyzed the distribution of the cells among the cell cycle phases after the various treatments (Figure 5). In these measurements, we distinguished four cell cycle phases; the resting G0/G1 phase, the synthesis S phase, the G2/M phase with cells having two sets of paired chromosomes, and the sub-G1 phase with dying cells. We found that the effect of the treatments was cell line dependent. In A375 cells, as expected, proliferation strongly decreased in the presence of vemurafenib (G2/M phase cells) (Figure 5C3), while HDACis alone had no effect. Vemurafenib induced cell death (sub-G1) in approximately 10% of the cells (Figure 5B3). This effect was further increased by combination with valproate but not with SAHA. HDACis alone only slightly increased cell death compared to control in this cell line. In A2058 cells, SAHA decreased significantly the number of cells in the G2/M phase, valproate alone did so to a lesser extent (Figure 5C3), but in combination with vemurafenib the effect was stronger. Treatment with SAHA alone and also in combination with vemurafenib increased the ratio of dying cells in this cell model (Figure 5C2). MEWO cells responded exclusively to SAHA treatment with decreased proliferation and none of the treatments induced cell death in these cells (Figures 5A2,A3). Based on these data, we conclude that HDACis alone decreased only modestly the viability and proliferation of melanoma cells, while combination treatments had a more substantial effect, although in a cell-type specific manner.

### HDACi Treatment Strongly Decreases the Migratory Activity of A375 Melanoma Cells in a PMCA4b-Dependent Manner

**Figure 6:**
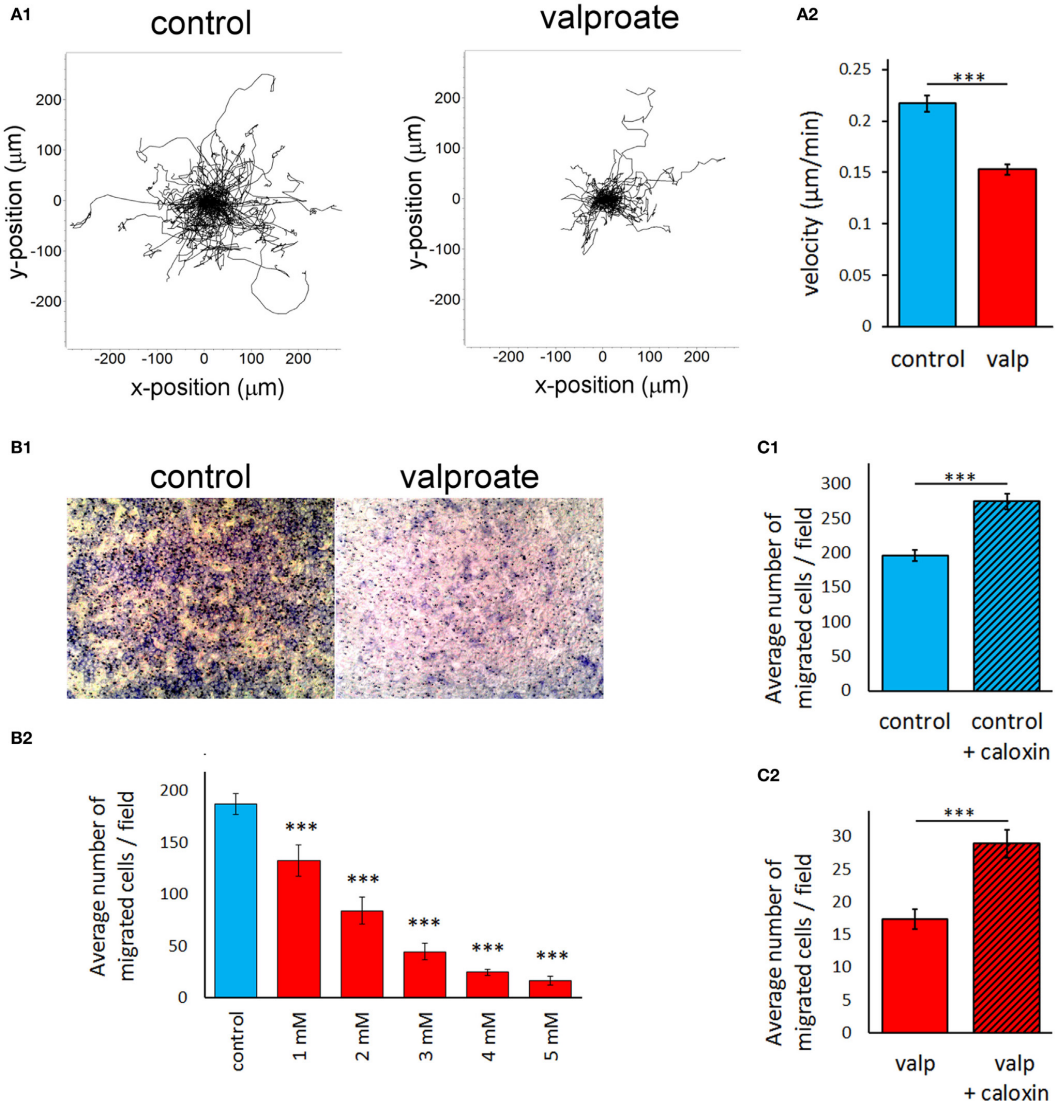
**Histone deacetylase inhibitor treatment strongly decreases the migratory activity of A375 melanoma cells in a PMCA4b-dependent manner**. A375 cells were treated with 4 mM valproate for 48 h before the migration analysis. **(A)** The random migratory activity of A375 melanoma cells was analyzed by automated fluorescence microscopy for 24 h (A1). Single cell migration trajectories of untreated, control cells (left graph) and valproate-treated cells (right graph) were recorded. Starting position of each cell trajectory was fixed to the origin of the plot. (A2) Mean velocity was evaluated for 24 h of migration. Data are shown as mean ± SEM of ≥100 individual cells of at least three independent measurements. Significance compared to control is denoted by ***p < 0.001; two-tailed unpaired *t*-test. **(B,C)**. Effect of valproate treatment of A375 cells **(B)** and specific inhibition of PMCA4 with caloxin 1c2 **(C)** on the directional migration of A375 cells. The effect of PMCA4 inhibition was studied on untreated (C1) and 4 mM valproate-treated (C2) A375 cells. The directional migration activity was assessed using Boyden chambers. B1 images show representative fields of view from the bottom of the filter (control and 4 mM valproate-treated A375 cells). B2 shows migration of cells in response to different concentrations (1–5 mM) of valproate. The cells that migrated through the membrane to the bottom face were counted. The bar graphs (B2, C1, C2) indicate the average number of migrated cells per field. Data are mean of three experiments ± SEM. Significance of comparisons is denoted by ***p < 0.001; two-tailed unpaired *t*-test.

We found previously that increased expression of PMCA4b in A375 cells resulted in decreased migratory activity. We assessed the effect of HDAC inhibition on both the random and directional migration of A375 cells (Figure 6). In all the experiments, the cells were treated with valproate (4 mM) for 48 h before the migration analysis. Random migration was recorded by using automated fluorescence microscopy (ImageXpress Micro XL) for 24 h. Representative trajectories of single cells after 48 h of valproate treatment clearly show that valproate strongly inhibited the migration of A375 cells (Figure 6A1). The velocity of valproate-treated cells decreased significantly compared to the untreated control cells (Figure 6A2). This effect was similar to that described previously for vemurafenib-treated A375 cells (21).

Directed migration of A375 cells was assessed using modified Boyden chamber assay (Figure 6B), in which fibronectin was used as a chemoattractant. Cells were allowed to migrate through the pores of the Boyden Chamber membrane for 6 h toward the fibronectin coat. We observed a sharp decrease in the directional movement of A375 cells pretreated with valproate, compared to that of the untreated cells (Figure 6B1). A half-maximal response of cells could be achieved at approximately 2 mM valproate treatment (Figure 6B2), which was comparable to that of the PMCA4b protein expression response to valproate under similar conditions (Figure 1A, middle panels). To evaluate the role of PMCA in this effect, we added the PMCA4-specific inhibitor, caloxin 1c2, to the cells immediately before the assay. Inhibition of PMCA4 resulted in a modest but significant increase in the directional migration activity in both the control and valproate-treated cells (Figure 6C) suggesting that PMCA4 activity is involved in the control of A375 cell motility.

## Discussion

Histone deacetylases are upregulated in many types of cancers (34) resulting in aberrant histone deacetylation that supposedly is associated with transcriptional repression of a variety of tumor suppressor genes. Previously, we identified PMCA4b as a putative metastasis suppressor in melanoma A375 cells that has the ability to suppress cell migration and metastasis without affecting cell proliferation (21). We also demonstrated that the expression of PMCA4b is enhanced in response to BRAF inhibition. In the current study, we show that the expression of this protein is under epigenetic control in melanoma cells.

Previously, it was demonstrated that in gastric and colon cancer cells short-chain fatty acid and trichostatin A treatment strongly increased the expression of PMCA4b and only moderately that of PMCA1b. Similar results were obtained after spontaneous differentiation of post-confluent cultures of these cells (10, 35). Furthermore, it has been shown that protein expression of PMCA4b is reduced in high-grade adenoma, colon cancer, and lymph node metastasis compared to normal mucosa, while the expression of PMCA1 remained unchanged (20). Similarly, in the breast cancer cell line, MCF-7 HDACi treatment markedly induced the expression of PMCA4b but not that of PMCA1b (9). In this work, we show that HDACi treatment induces significantly the abundance of PMCA4b in melanoma cells, irrespective of their BRAF mutational status but in a cell-type-dependent manner. Furthermore, a substantial increase in the abundance of the housekeeping isoform, PMCA1was also seen in the two BRAF-mutant cell lines, A375 and A2058, but not in BRAF wild-type MEWO cells. We demonstrated that in A375 cells the enhanced PMCA4b level increased Ca^2+^ removal from the cytosol, which coincided with a decreased migratory potential of these highly motile cells. Selective inhibition of PMCA4b activity partially reversed the effect of HDAC inhibition on both the Ca^2+^ signal and the directional movement of A375 cells, providing additional evidence that PMCA4b has a primary role in the regulation of cell motility in this cell line.

Another important finding of the current study is that combination of the two drugs, HDACi and vemurafenib, did not result in any substantial additional effect on the PMCA expression. These data suggest that in these melanoma cell lines the expression of both PMCA1 and PMCA4b is downregulated by epigenetic factors. One possible explanation for the lack of additivity between BRAF and HDAC inhibition on PMCA4b expression is that the effect of ERK inhibition could itself be indirect and be mediated by histone acetylation (36). Although vemurafenib treatment did not affect H3 histone acetylation in the melanoma cell lines used in our studies, it is still conceivable that acetylation of other histones is responsible for the regulation of PMCA4b expression by vemurafenib.

It is possible that decreased expression of PMCAs might contribute to the maintenance of an elevated intracellular calcium level. In contrary, increased expression of several types of Ca^2+^ channels was identified in both melanoma cells and melanoma tissues, and the inhibition of these channels led to increased cell death and decreased metastatic activity. Transient receptor potential cation channels (TRPM1, 2,7 and 8) have been shown to be upregulated in melanomas (22), where they contributed to increased proliferation and metastatic capacity. In addition, elevated expression of the store-operated Ca^2+^ channels, ORAI1 and ORAI2, and of the stromal interacting molecule 1 and 2 (SOCE) was also described. Inhibition of SOCE decreased both melanoma cell proliferation and migration (23, 24).

Previously, we demonstrated that PMCA activity can reduce the SOCE-mediated Ca^2+^ signal (37) and most recently that PMCA4b can inhibit melanoma cell migration. A direct role of PMCA4b in the regulation of migration was also demonstrated in VEGF-treated endothelial cells (38), which involved the NFAT pathway. During collective migration of human umbilical vein endothelial cells (39), PMCA4b showed polarized localization, necessary to maintain the intracellular Ca^2+^ gradient characteristic of directionally migrating cells.

In the current study, we demonstrated that in melanoma cells PMCA4b expression is regulated through histone acetylation. Upregulation of PMCA4b abundance through the inhibition of HDACs resulted in a significant decrease in cell motility of the highly invasive A375 cells. HDACis had a similar effect to what was shown previously with vemurafenib treatment. While HDAC inhibition affects the expression of a broad range of proteins, the specific role of PMCA4b in the regulation of cell migration of A375 cells was confirmed by the reverting effect of the PMCA4b-specific inhibitor, caloxin 1c2. It is also important to note that the expression of the SOCE channels (STIM or ORAI) was not affected by the treatments, indicating that SOCE could not be responsible for the suppressed migratory capacity of the HDACi-treated cells.

At the transcriptional level, PMCA1 is under tight control. Vitamin D is one of its known regulators which has been shown to induce PMCA1 expression effectively in the small intestine, kidney distal tubules, and osteoblasts (40). More recently, PMCA1 upregulation was observed in colon cancer cells (41). Targeted deletion of PMCA1 in mice resulted in impaired responsiveness to 1,25-dihydroxyvitamin D_3_, the active metabolite of vitamin D, and in decreased bone mineral density (42). Recent clinical studies have suggested that melanoma patients with lower levels of vitamin D may have poorer survival (43, 44). As vitamin D exerts its gene regulatory effect by attracting HDACis to the promoter region of target genes, our results suggest that vitamin D might regulate PMCA1 expression by epigenetic mechanisms.

The SERCAs play an important role in the regulation of the intracellular Ca^2+^ homeostasis. There is not much known, however, about their role in cell migration. SERCA2 has been shown to decrease the migratory capacity of dendritic cells in response to the chemokine CCL21 (45), while it was demonstrated that nitric oxide-mediated activation of SERCA2b is necessary for VEGF-induced endothelial cell migration (46). We found that in A375 cells only SERCA2, the housekeeping isoform, was present and its expression was decreased by treatment with SAHA but was not effected by valproate. Since in both of our migration model systems we used valproate treatment, it is unlikely that SERCA2 affected the migratory capacity of these cells.

As it has been shown previously that HDAC inhibition induced apoptosis in various cancer types, we investigated the effect of SAHA, valproate, and vemurafenib on the viability and cell cycle progression of these three melanoma cell lines. We found that viability was not affected substantially in any of the cell lines tested, while the ratio of proliferating cells was altered in a cell-type-dependent manner. A375 cells, which are highly sensitive to the BRAF inhibitor, vemurafenib, reacted more efficiently to vemurafenib treatment than to either of the HDACis alone. Combination of the two types of drugs could further increase the number of cells in the sub-G1 phase. In A2058 cells, which carry also a mutation in the tumor suppressor PTEN, vemurafenib had only a small effect on the number of cells in the G2/M phase; however, SAHA and valproate in combination with vemurafenib could decrease their ratio significantly. The moderate effect on cell death in our treatments is in good accordance with the results of others (47), who found strong increase in cell death only at much higher concentration of HDACis (13).

Overall, our data show that the HDACis, SAHA and valproate, enhanced the expression of the previously recognized metastasis suppressor PMCA4b in an ERK-independent manner and reduced the cell motility without having substantial influence on cell proliferation. Specific inhibition of PMCA4b activity partially blocked the effect of HDACis underlining the role of PMCA4b in the regulation of cytosolic Ca^2+^ concentration and cell motility. Moreover, the HDACis also enhanced the expression of the housekeeping PMCA1 isoform in the BRAF-mutant cells, suggesting an even more complex role of the PMCAs in the Ca^2+^ homeostasis of melanoma cells. Our results support the further evaluation of HDACis alone or in combination with other drugs for the treatment of metastatic melanoma.

## Author Contributions

LH conceived, designed and performed experiments, analyzed and interpreted the data, was involved in writing the paper. RP designed and supervised the non-directional and Boyden chamber migration experiments. She analyzed and interpreted the data of these experiments. She prepared the related chapters and figures of the manuscript. JM maintained cell culture and performed treatments and Western blot analysis. KP performed Ca^2+^ signaling experiments and analyzed the data. KV developed methodology. IK supervised the Boyden chamber experiments. ES maintained A375 cell culture and carried out the Boyden chamber experiments. MW carried out and analyzed the qPCR experiments. MG supervised the qPCR experiments and was involved in writing the paper. ZH carried out the non-directional migration experiments and analyzed the data of these experiments. LHom supervised the non-directional migration experiments and read the paper. CA was involved in helpful discussions and critical reading of the paper. GT developed methodology. BH was involved in interpreting the data and read the paper. JT was involved in helpful discussions and critical reading of the paper. EK was involved in interpreting the data and writing the paper. AE conceived and designed the experiments, analyzed and interpreted the data, involved in writing the paper, and supervised the study.

## Conflict of Interest Statement

The authors declare that the research was conducted in the absence of any commercial or financial relationships that could be construed as a potential conflict of interest.
