# Supplementary material for: Histone Deacetylase Inhibitor Treatment Increases the Expression of the Plasma Membrane Ca2+ Pump PMCA4b and Inhibits the Migration of Melanoma Cells Independent of ERK
Source: Front Oncol. 2017 May 24;7:95. doi: 10.3389/fonc.2017.00095 (PMC5442207; doi:10.3389/fonc.2017.00095)
Supplement: Supplementary file 1 [file data_sheet_1.docx]

Supplementary Material

Histone deacetylase inhibitor treatment increases the expression of the plasma membrane Ca^2+^ pump PMCA4b and inhibits migration of melanoma cells independent of ERK

**Luca Hegedüs^1,8^, Rita Padányi^2^, Judit Molnár^2^, Katalin Pászty^3^, Karolina Varga^2,4^, István Kenessey^2^, Eszter Sárközy^2^, Matthias Wolf^5^, Michael Grusch^5^, Zoltán Hegyi^6^, László Homolya^6^, Clemens Aigner^1^, Tamás Garay^7^, Balázs Hegedüs^1,7^, József Tímár^2,7^ , Enikö Kállay^8^, Ágnes Enyedi^2,7*^**

**^1^Department of Thoracic Surgery, Ruhrlandklinik, University Clinic Essen, Essen, Germany**

**^2^2^nd^ Institute of Pathology, Semmelweis University, Budapest**

**^3^Molecular Biophysics Research Group of the Hungarian Academy of Sciences and Department of Biophysics, Semmelweis University, Budapest, Hungary**

**^4^MTA-SE-NAP Brain Metastasis Research Group of the Hungarian Academy of Sciences and Semmelweis University, Budapest, Hungary**

**^5^Institute of Cancer Research, Department of Medicine I, Comprehensive Cancer Center Vienna, Medical University of Vienna**

**^6^Institute of Enzymology, Research Centre for Natural Sciences, Hungarian Academy of Sciences, Budapest, Hungary**

**^7^Molecular Oncology Research Group of the Hungarian Academy of Sciences and Semmelweis University, Budapest, Hungary**

**^8^Department of Pathophysiology and Allergy Research, Comprehensive Cancer Center Vienna, Medical University of Vienna**

*** Correspondence:** Agnes Enyedi, Email: [enyedi.agnes@med.semmelweis-univ.hu](mailto:enyedi.agnes@med.semmelweis-univ.hu)

# Supplementary Figures and Tables

## Supplementary Figures


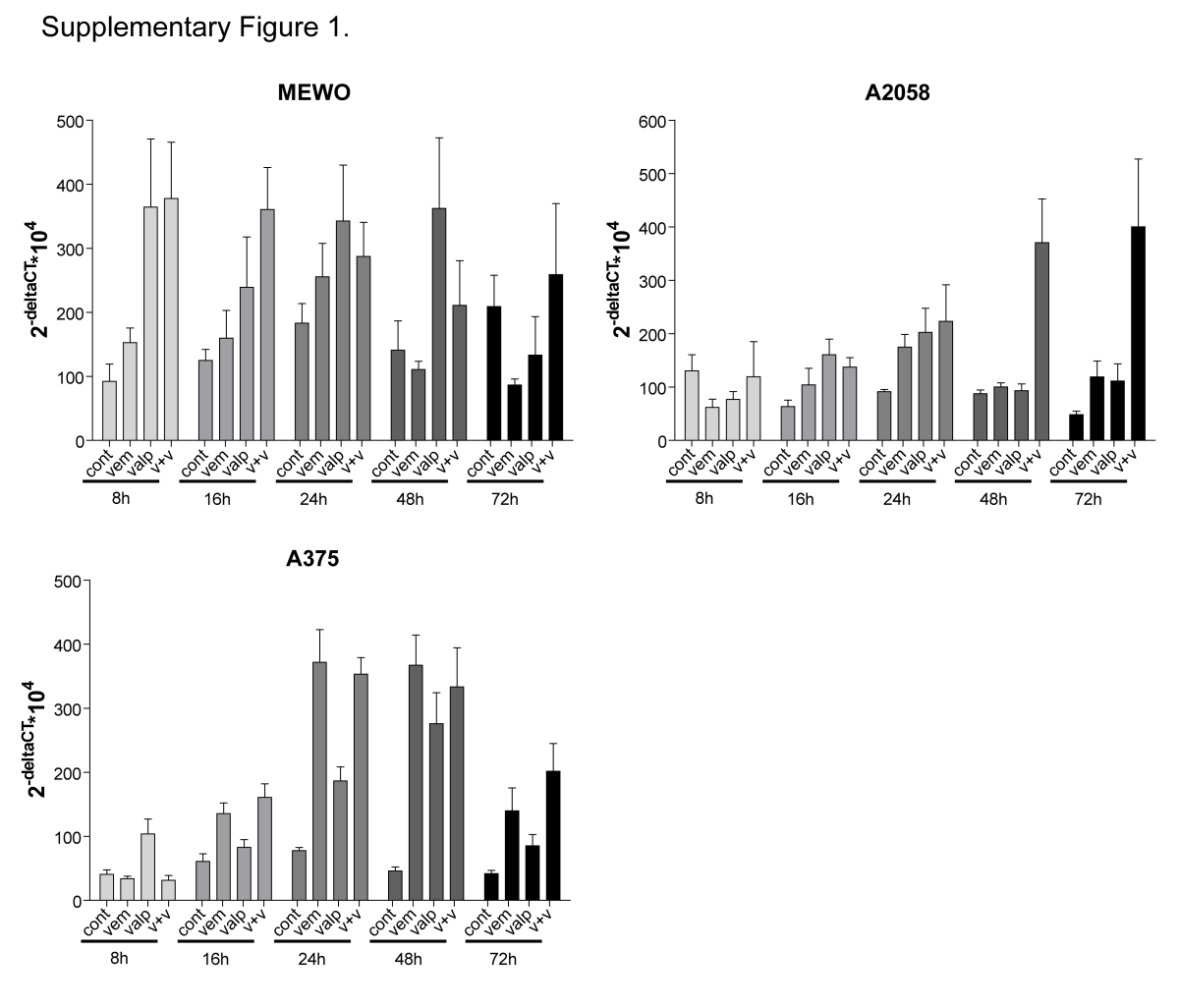


**Supplementary Figure 1.** Effect of HDAC inhibitor treatment and BRAF inhibition alone and in combination on mRNA expression of PMCA4b in melanoma cells. Expression of PMCA4b was analyzed by quantitative real-time PCR after treatment with either 2mM valproate or 0.5 μM vemurafenib alone or in combination in MEWO, A375 and A2058 cell lines.


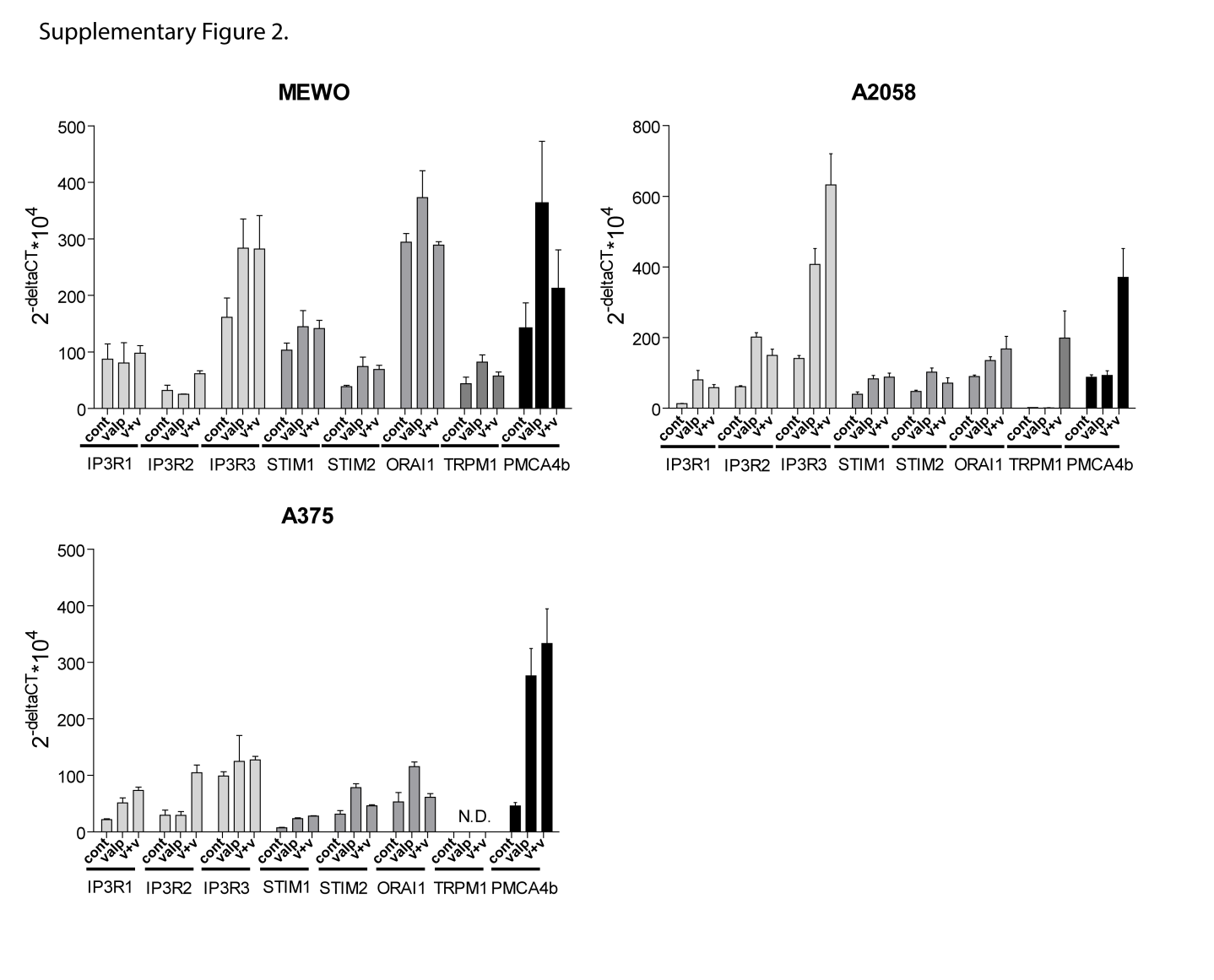


**Supplementary Figure 2.** Changes in the mRNA level of Ca^2+^ channels was analyzed after treatment with valproate (2 mM) alone or in combination with vemurafenib (0.5 μM). Expression of inositol 1,4,5-trisphosphate receptor type 1-3 (IP3R1, IP3R2, IP3R3), ORAI calcium release-activated calcium modulator 1 (ORAI1), stromal interaction molecule 1 and 2 (STIM1, STIM2), transient receptor potential cation channel subfamily M member 1 (TRPM1) was measured by quantitative real-time PCR analysis. Expression was normalized to glyceraldehyde-3-phosphate dehydrogenase (GAPDH) and bars represent means and S.E.M. of three independent experiments performed in duplicates. N.D., not detectable.


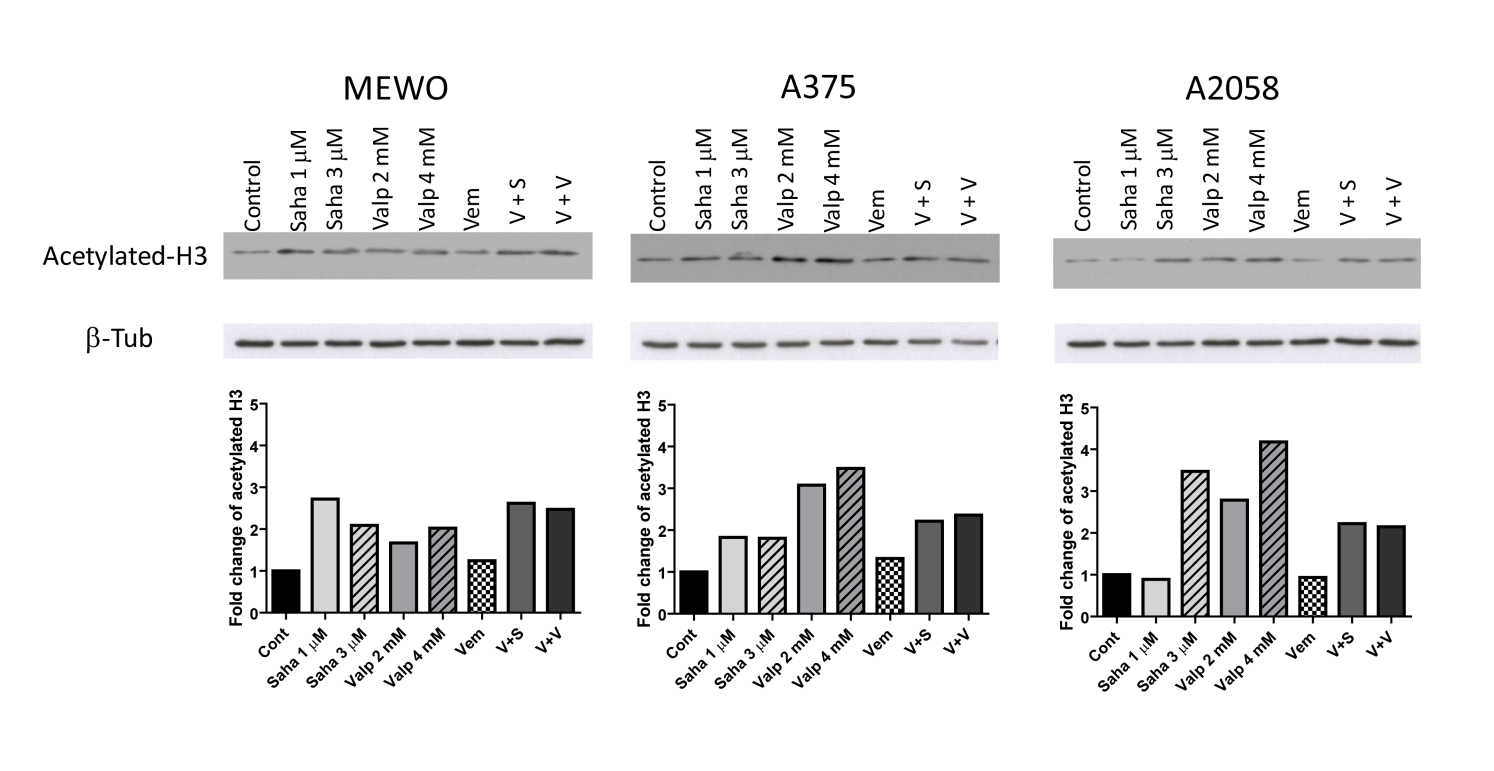


**Supplementary Figure 3.** Changes of acetylated histone H3 protein level after treatment with HDAC inhibitors alone or in combination with vemurafenib (vem, vemurafenib (0.5 μM); S, SAHA (1μM); V, Valproate (2 mM)) for 48 hours. Western blots were analyzed by densitometry. Data were normalized to the expression levels of β-tubulin and changes in acetylated-H3 protein level were expressed as fold increase over the untreated controls.
